# Proline/serine‐rich coiled‐coil protein 1 inhibits macrophage inflammation and delays atherosclerotic progression by binding to Annexin A2

**DOI:** 10.1002/ctm2.1220

**Published:** 2023-03-17

**Authors:** Hangyu Pan, Zhongzhou Guo, Ping Lv, Kexin Hu, Tongwei Wu, Zixiang Lin, Yazhi Xue, Yanan Zhang, Zhigang Guo

**Affiliations:** ^1^ Department of Cardiology State Key Laboratory of Organ Failure Research Nanfang Hospital Southern Medical University Guangzhou China; ^2^ Department of Pharmacy Zhujiang Hospital Southern Medical University Guangzhou China; ^3^ Department of Cardiovascular Surgery Nanfang Hospital Southern Medical University Guangzhou China; ^4^ Department of Medicine Ultrasonics Nanfang Hospital Southern Medical University Guangzhou China; ^5^ Department of Cardiology Shenzhen Hospital Huazhong University of Science and Technology Union Shenzhen China; ^6^ Department of General Practice Nanfang Hospital Southern Medical University Guangzhou China; ^7^ Department of Cardiology Huiqiao Medical Center Nanfang Hospital Southern Medical University Guangzhou China

**Keywords:** Annexin A2, atherosclerosis, inflammation, macrophage, proline/serine‐rich coiled‐coil protein 1

## Abstract

**Background:**

Atherosclerosis (AS), the main pathological basis of life‐threatening cardiovascular disease, is essentially caused by chronic macrophage inflammation. Overexpression of proline/serine‐rich coiled‐coil protein 1 (PSRC1) reduces macrophage inflammatory responses and delays AS development. However, the exact mechanism of PSRC1 is unclear.

**Methods:**

Proteins interacting with PSRC1 were screened by proteomics in RAW264.7 cells, followed by RT‒qPCR, immunoprecipitation and immunofluorescence to explore the specific mechanistic pathways affecting inflammation. CRISPR‒Cas9 constructs for PSRC1^−/−^ApoE^−/−^(DKO) mice and high‐fat diet‐fed ApoE^−/−^ and DKO mice were used for AS models for in vivo experiments. Upstream transcription factors of PSRC1 were predicted by ATAC‐seq, ChIP‐seq and UCSC, and the regulatory mechanism was verified by ChIP‒qPCR and dual luciferase assays. Peripheral blood serum and monocytes were collected from coronary artery disease (CAD) patients and non‐CAD patients.

**Results:**

Increased binding of ANXA2 to PSRC1 in macrophages under oxidized low‐density lipoprotein stimulation and decreased release of ANXA2 to the extracellular compartment were observed. Knockdown of ANXA2 in AS model mice delayed AS progression. Knockdown of ANXA2 in DKO mice reversed the AS‐promoting effect of PSRC1 knockdown. Mechanistically, ANXA2 promotes STAT3 phosphorylation, which in turn promotes inflammatory responses. In addition, SP1 is a PSRC1 upstream repressive transcription factor, and the SP1 inhibitor mithramycin (Mith) elevated PSRC1 expression and exerted anti‐AS effects in AS model mice. Patients with CAD had considerably greater serum levels of ANXA2 than those without CAD, and Mith reduced the secretion of ANXA2 in peripheral blood monocytes of CAD patients.

**Conclusion:**

In macrophages, PSRC1 can interact with ANXA2 to inhibit its extracellular release and delay AS development. SP1 is an upstream transcription factor of PSRC1 and inhibits the transcription of PSRC1. The SP1 inhibitor Mith can elevate PSRC1 levels and slow AS progression while reducing ANXA2 release from monocytes in CAD patients. Mith is expected to be a new agent for AS treatment.

## Graphical abstract

- Through ANXA2, PSRC1 in macrophages affects the development of AS, while ANXA2 influences macrophage inflammation by regulating STAT3 phosphorylation.
- SP1 inhibitor mithramycin increases PSRC1 expression in macrophages and delays AS through PSRC1, since SP1 is a PSRC1 repressor transcription factor.
- Monocytes from CAD patients decrease ANXA2 secretion when treated with mithramycin.

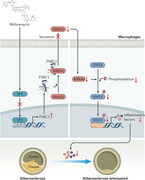

## 1 INTRODUCTION

Atherosclerotic cardiovascular disease (ASCVD) is the primary cause of death from cardiovascular disease, which is a serious hazard to human health.^1^ In 2019, 17.9 million fatalities worldwide were attributed to cardiovascular disease, which also contributed to 32% of all deaths globally and 38% of the 17 million non‐communicable disease‐related premature deaths in persons under the age of 70 that were reported.^2^ Currently, both primary and secondary strategies for preventing ASCVD are focused on correcting abnormal serum cholesterol levels, but studies have confirmed that even when aggressive and intensive lipid‐lowering therapy reduces low‐density lipoprotein cholesterol (LDL‐C) to very low levels, the occurrence of major cardiovascular events decreases by only approximately 30%.^3^, ^4^ Atherosclerosis (AS) is an inflammatory disease, and the inflammatory response has a role in AS development, according to numerous studies; additionally, clinical studies have demonstrated that anti‐inflammatory therapy can achieve significant clinical effects and reduce the occurrence of cardiovascular events.^5^ Macrophages are the central cell type in the inflammatory response and the primary cells involved in foam cell formation during the development of AS^6^; therefore, finding new targets that can affect the macrophage inflammatory response could provide novel strategies to prevent and treat AS.

Proline/serine‐rich coiled‐coil protein 1 (PSRC1), which is a microtubule‐associated protein, is an essential molecule for mitotic progression in normal eukaryotic cells. In 2007, Samani published a genome‐wide association study on coronary artery disease (CAD), and this study first identified the association of PSRC1 gene polymorphisms with the development of CAD.^7^ Subsequently, later studies confirmed the association of PSRC1 gene polymorphisms with the development of AS in different ethnic populations.^8^, ^9^, ^10^ Moreover, quantitative trait locus analysis of gene expression revealed a negative correlation between PSRC1 gene expression levels and the occurrence of CAD.^11^ In our previous study, overexpression of PSRC1 significantly reduced aortic plaque areas, increased plaque stability, and decreased serum inflammatory factor levels in ApoE^−/−^ mice. Additionally, overexpression of PSRC1 in macrophages significantly reduced inflammatory factor expression and secretion.^12^ However, exactly how PSRC1 regulates macrophage inflammatory responses has not been clearly demonstrated.

Annexin A2 (ANXA2) is a member of a family of membrane‐associated proteins, and it is characterized by its ability to bind to calcium ions and thus participate in a series of calcium‐dependent membrane biological activities. ANXA2 is widely expressed in various parts of the body; the level of ANXA2 expression varies widely in different tissues and cells, and it is expressed at high levels mainly in monocytes and macrophages.^13^ Moreover, ANXA2 is widely expressed in the nucleus, cytoplasm, and extracellular space and can both perform intracellular membrane‐related physiological functions and be released to function in the extracellular space. The role played by ANXA2 in AS has not been elucidated. According to a proteomic analysis, ox‐LDL stimulation dramatically boosted PSRC1 binding to ANXA2 in macrophages. Our study confirmed that PSRC1 exerts proinflammatory effects by increasingly binding to ANXA2 and thus inhibiting its release into the extracellular space, ultimately delaying AS progression.

## 2 METHODS

### 2.1 Study population

The trial population consisted of 200 patients with CAD and 80 individuals without CAD, and the inclusion criteria were as follows: (1) CAD patients: (1) 18–75 years old, no restriction on sex, and (2) patients met the diagnosis of coronary atherosclerotic heart disease in China (at least one major coronary artery or its major branches had ≥50% internal diameter stenosis); (2) non‐CAD patients: (1) 18–75 years old, no restriction on sex, and (2) patients did not meet the diagnostic criteria of CAD, and coronary angiography indicated that the internal diameter stenosis of any major coronary artery or its major branches was <50%. Exclusion criteria were as follows: inflammatory diseases of the heart such as severe myocarditis, pericarditis, and endocarditis, severe hepatic and renal insufficiency, infectious diseases, haematological disorders, tumours and autoimmune system diseases. All the patients understood the whole process of the trial and showed good compliance. They signed the informed consent form and consented to participate in the trial together with their families.

### 2.2 Ethics

The Ethics Committee of Nanfang Hospital, Southern Medical University approved the present study (NFEC‐2017‐K3‐Revision02), and written informed consent was obtained from all the subjects. All procedures conformed to the principles outlined in the Declaration of Helsinki.

The animal protocols were approved by the Animal Experiment Committee of Nanfang Hospital at Southern Medical University (Guangzhou, China).

### 2.3 Monocyte isolation

The density gradient technique was used to separate PBMCs from whole blood (Ficoll Plus, BD Biosciences). Monocytes were isolated by adherence to plastic and cultured using RPMI 1640 + 10% foetal bovine serum (FBS) in a humidified incubator at 37°C with 5% CO_2_. After 24 h of stimulation with 200 nM Mith (Sigma, USA), the cells were used for further experiments.

### 2.4 Generation of recombinant adenoviruses

The DNA sequence for the green fluorescent protein (GFP) and the mouse ANXA2 were subcloned into an adenovirus shuttle plasmid vector (pAdM‐U6‐MCS‐mCMV‐GFP‐SV40pA) carrying a cytomegalovirus promoter using the sequence from GenBank. As a comparison, a recombinant adenovirus that encodes enhanced GFP (Ad‐GFP) was used.

### 2.5 Generation of recombinant adeno‐associated virus

The C‐6944 scAAV.U6.shRNA (mAnxa2).CAG.EGFP.WPRE.SV40pA plasmid vector was used to clone the ANXA2 cDNA after it had been amplified by PCR. Sequencing was used to confirm the ANXA2 gene's correct sequence in this construct.

### 2.6 Animal model

All diets, including the normal chow diet and the high‐fat diet, were acquired from Guangdong Medical Laboratory Animal Center (Guangdong, China). ApoE^−/−^ mice with a C57BL/6 J background were obtained from Viewsolid Biotech Co., Ltd. (Beijing, China). PSRC1^−/−^ mice were generated at Viewsolid Biotech Co., Ltd. using the CRISPR–Cas9 system to delete exon 4 of PSRC1 in ApoE^−/−^ mice.

AS model: Mice were fed on a chow diet until 8 weeks of age and were given a HFD for 12 weeks.

Ad‐GFP and Ad‐shANXA2 model: After 12 weeks of HFD feeding, Ad‐GFP or Ad‐shANXA2 at a dose of 5 × 10^8^ pfu was injected into the tail vein. HFD feeding was continued for 2 weeks, the virus was administered once more through the tail vein, and HFD feeding was continued for 2 weeks.

AAV‐GFP and AAV‐shANXA2 model: After 12 weeks of HFD feeding, AAV‐GFP or AAV‐shANXA2 at a dose of 2 × 10^11^ pfu was injected into the tail vein. HFD feeding was continued for 2 weeks, the virus was administered once more through the tail vein, and HFD feeding was continued for 2 weeks.

Mith model: After 12 weeks of HFD feeding, PBS or Mith (150 μg/kg/d) was administered via intraperitoneal injection for 4 consecutive weeks, during which time HFD feeding was continued.

The mice were anaesthetized by a mixture of 2%–5% inhaled isoflurane with oxygen and air. The mice were sacrificed by cervical dislocation after the proper level of anesthesia was achieved, and the tissues were collected.

### 2.7 Atherosclerotic lesion analysis

After a mouse was executed, the aorta was retained after systemic perfusion of the left ventricle, and the portion from the ascending aorta to the iliac artery was harvested and longitudinal dissection, stained with Oil Red O and photographed against a blue background using a digital camera. Oil Red O was used to stain frozen sections of aortic root tissue. In addition, paraffin sections were generated after fixation using formalin and subjected to HE staining (to identify the necrotic core areas) and Masson's trichrome (to identify collagen fibres). Immunofluorescence staining of the aortic root tissue was performed using anti‐CD68 (to identify macrophages, CST) and anti‐ANXA2 (CST) antibodies. Immunohistochemical staining was performed using anti‐α‐SMA (to identify vascular smooth muscle cells [SMCs], CST). Images were obtained using microscope (Olympus, Japan) and confocal laser scanning microscope (LEICA, Germany).

### 2.8 Plasma lipids analysis

Animals were anaesthetized, and blood was taken from the eyes. The top layer of plasma was taken after centrifuging peripheral blood for 15 min at 3000 rpm, and the measurements of triglyceride (TG), cholesterol (CHOL), LDL and HDL using assay kits (Nanjing Jiancheng Bioengineering Institute, China).

### 2.9 Peritoneal macrophage isolation

After the mice were sacrificed, 5 mL of RPMI 1640 (Gibco, USA) containing antibiotics was injected into the peritoneal cavity, the medium was massaged for 5 min and then withdrawn, centrifuged at 1000 rpm for 10 min, resuspended in RPMI 1640+10%FBS and spread in a culture dish, and the adnexal cells were taken as peritoneal macrophages.

### 2.10 Cell culture and transfection

THP‐1 cells (Zhuosheng Biotech, China) and Raw264.7 cells (China Center for Type Culture Collection, China) were cultured in RPMI 1640+10% FBS and DMEM (Gibco, USA) +10%FBS, respectively, in an incubator at 37°C and 5% CO_2_. When the Raw264.7 cells had grown to 40%–50% confluence, using Lipofectamine RNAimax (Invitrogen, USA), the cells were transfected with ANXA2 siRNA (Jikai Biotech, China).

For adenovirus transfection, after being diluted to the proper MOI with culture medium, the viral stock solution was introduced directly to the medium. Cell culture supernatants were collected 48–72 h after transfection for the next step of the experiments.

### 2.11 Cell immunofluorescence staining

When Raw264.7 cells reached 50%–60% confluence in confocal dishes, immunofluorescence staining was performed using an anti‐p‐STAT3 (CST), anti‐STAT3 (CST), anti‐ANXA2 (Santa Cruz) and anti‐PSRC1 (Solarbio). The secondary antibody was purchased from Fude Biotech. Images were acquired using a Leica LAS X (Leica, Germany).

### 2.12 Non‐labelled quantitative proteomics

RAW264.7 cells were divided into the Ad‐GFP and Ad‐PSRC1 groups and treated with 100 μg/mL ox‐LDL for 24 h. The cells were lysed, and the lysates were subjected to pull‐down assays using anti‐FLAG M2 magnetic beads (Millipore, USA). The obtained products were analysed by quantitative mass spectrometry with data independent acquisition (DIA) using an ultrahigh resolution triplet mass spectrometer (Thermo ScientificTM Orbitrap FusionTM TribridTM). First, the samples were subjected to reductive alkylation and trypsin digestion; then, the peptides were digested to remove the nonvolatile salts and subjected to data‐dependent acquisition and DIA; finally, the data were imported into Skyline software (Skyline v4.2) for quantitative analysis. Proteins whose expression was up‐regulated by more than 1.2‐fold in the Ad‐PSRC1/Ad‐GFP groups and with a false discovery rate (FDR) <.05 were considered proteins with increased PSRC1 interactions.

### 2.13 ATAC‐seq

ATAC‐Seq were performed on 50 000 THP‐1 cells. The cells were lysed using cold lysis buffer and then suspended in 1X reaction buffer (NovoNGS Tn5 Transposase, Shanghai, China). Transposase reactions were carried out at 37°C, and DNA was purified using a MinElute PCR Purification Kit (QIAGEN, Germany). The generated DNA fragments were amplified by two sequential 6‐cycle PCRs. The final PCR products were purified and quantified by a MinElute PCR Purification Kit prior to sequencing. Subsequent sequencing and data analysis were performed by Saicheng Biotechnology Co., Ltd. (Guangzhou, China).

### 2.14 Chromatin immunoprecipitatio‐qPCR and sequencing (ChIP‒qPCR and ChIP‐seq)

When THP‐1 cells had grown to a population size of 4 × 10^7^, 200 nM Mith was added to the experimental group. A ChIP Kit (ThermoFisher, USA) was used to complete the next experiments. Formaldehyde was used to cross‐link the cells, with a final concentration of 1%. The addition of 1x glycine solution halted the cross‐linking reaction. Cells were lysed using Lysis Buffer 1 supplemented with protease inhibitor, and then the supernatants were removed and resuspended in MNase Digestions Buffer working solution. Then, micrococcal nuclease was added, and the reactions were stopped by adding MNase stop solution after incubation at 37°C. The supernatants were removed, and the precipitates were resuspended in lysis buffer 2 with protease/phosphatase inhibitor, incubated on ice, centrifuged and transferred to new precooled 1.5‐mL centrifuge tubes. Five microlitres of the supernatants from the above step were stored at −20°C, and used as the input. Forty‐five microlitres of the supernatants were placed into centrifuge tubes containing 1 × IP Dilution Buffer. Then, diluted lysate was added to the plug spin column, incubated with anti‐SP1 antibodies (Abcam, UK), and mixed by inversion overnight at 4°C. Following immunoprecipitation, the beads were recovered using a magnet, and the DNA was uncrosslinked by incubation with proteinase K at 65°C. Purifying and preparing the acquired DNA for qPCR and sequencing.

### 2.15 Dual‐luciferase reporter assay

The SP1‐CDS region was cloned into the pcDNA3.1 plasmid, and the wild‐type and mutant PSRC1 promoter regions were cloned into the luciferase vector PGL‐3. After plasmid amplification, the plasmids were extracted using the Plasmid Extraction Kit (QIAGEN, Germany). When THP‐1 cells had grown to a population size of 1×10^6^, the plasmids were transfected into cells using Lipofectamine 3000 (Invitrogen, USA), and fluorescence values were measured using a UV spectrophotometer 24–48 h after transfection.

### 2.16 RT‐qPCR

Total RNA was extracted from Raw264.7 cells using TRIzol reagent (Accurate Biology, China). cDNA was synthesized using 5× Evo M‐MLVRT Master Mix (Accurate Biology, China). RT–qPCR was performed on a LightCycler 480 II (Roche, Switzerland) using 2×SYBR Green Pro Taq HS Premix (Accurate Biology, China). Relative gene expression was calculated using the 2^−ΔΔCt^ method, with β‐actin serving as an internal reference control. The primers could be found in Table S3.

### 2.17 Western blotting analysis

THP‐1 or Raw264.7 cells were lysed with RIPA buffer mixed with protease and phosphatase inhibitors (100:1). Protein concentrations were quantified. Sodium dodecyl sulfate‐polyacrylamide gel electrophoresis (SDS‐PAGE) were used to separate equal amounts of protein, which were then transferred to PVDF membranes (Millipore, Burlington, MA). After being blocked in 5% skimmed milk powder for 1 h at room temperature, the membranes were treated with the following primary antibodies overnight at 4°C: anti‐SP1 (Abcam), anti‐PSRC1 (GeneTex), anti‐ANXA2 (CST), anti‐p‐STAT3 (CST), anti‐STAT3 (CST), anti‐ERK1/2 antibody (Abcam), anti‐p‐ERK1/2 (Abcam), anti‐P38 (CST), anti‐p‐P38 (Proteintech) and anti‐β‐tubulin (Fude Biotech). The membranes were incubated wit secondary antibodies (Fude Biotech). Using an ECL kit, the protein bands were seen (Affinity, China) and analysed by ImageJ (National Institutes of Health, USA).

### 2.18 Coimmunoprecipitation

Once cells had grown to 60%–70% confluence, total protein was extracted using RIPA buffer mixed with protease inhibitors after treatment and incubated overnight with an anti‐ANXA2 antibody (Abcam, UK) and IgG. Then, the cells were immunoprecipitated with magnetic beads and incubated overnight at 4°C. Finally, the beads were eluted, SDS‒PAGE loading buffer was added and incubated at 95°C to denature the proteins, and the target protein levels were measured by Western blotting analysis.

### 2.19 Enzyme‐linked immunosorbent assay (ELISA)

Whole blood specimens were collected from animals and patients and centrifuged at 3000 rpm for 15 min. The upper serum layer was separated and stored frozen at −80°C. The levels of IL‐1β, IL‐6 and ANXA2 in animal and patient samples were measured by ELISA kits (Elabscience, China).

### 2.20 Statistical analysis

Comparisons of normally distributed data between two groups were performed using Student's *t* test, and data from several groups were compared using one‐way ANOVA (analysis of variance). The Mann‒Whitney *U* rank sum test was used for comparisons of non‐normally distributed data between two groups. Statistical data are expressed as percentages of different groups, and the chi‐square test was employed to compare differences between the two groups. The mean ± SD (standard deviation) is used to express all of the data. GraphPad Prism v8.4 was used for statistical analysis. Baseline analysis and regression analysis were performed using SPSS v26.0. It was considered statistically significant at *p* < .05.

### 2.21 Role of the funding source

The sponsors of funding were independent from study design, data collection, data analyses and writing of paper.

## 3 RESULTS

### 3.1 PSRC1 binds to ANXA2 and reduces its extracellular release

**FIGURE 1:**
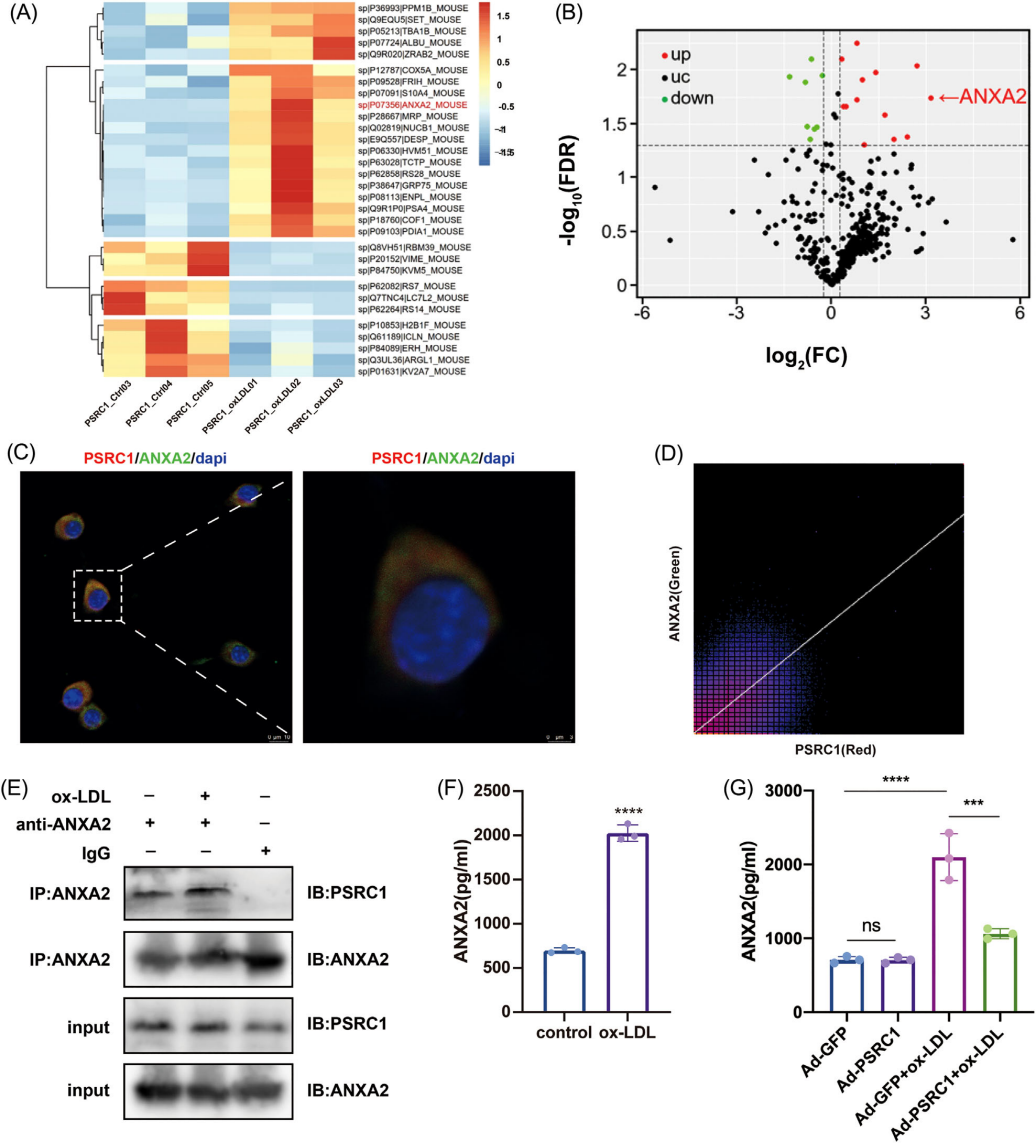
PSRC1 binds to ANXA2 and reduces its extracellular release. (A) Thermogram of Ad‐PSRC1+ox‐low‐density lipoprotein (LDL) versus Ad‐green fluorescent protein (GFP)+ox‐LDL quantitative proteomics FDR < .05 protein. (B) Ad‐PSRC1+ox‐LDL versus Ad‐GFP+ox‐LDL quantitative proteomics volcano plot. Red dots represent proteins with FDR < .05 and up‐regulated fold change of more than 1.2‐fold, green dots represent proteins with FDR < .05 and down‐regulated fold change of more than 1.2‐fold, black dots represent proteins with FDR > .05. (C and D) Immunofluorescence staining and quantification via ImageJ showed the colocalization of ANXA2 with PSRC1 in Raw264.7 cells. Left: scale bar = 10 μm. Right: scale bar = 3μm. (E) Raw264.7 cells were treated with 100 μg/mL ox‐LDL or PBS and then immunoprecipitated using an anti‐ANXA2 antibody. The interacting proteins were detected by Western blotting. +: positive control; −: negative control. (F) ANXA2 concentrations in the supernatants of Raw264.7 cells treated with 100 μg/mL ox‐LDL or PBS for 24 h were measured by ELISA, (2027.23 ± 93.55) pg/mL versus (697.01 ± 30.08) pg/mL, *n* = 3 per group, Student's *t* test, ****p < .0001. (G) ANXA2 concentrations in the supernatants of cells transfected with Ad‐PSRC1 or Ad‐GFP and treated with 100 μg/mL ox‐LDL for 24 h were measured by ELISA, (1061.65 ± 68.52) pg/mL versus (2098.67 ± 318.41) pg/mL, *n* = 3 per group, one‐way ANOVA, ***p < .001. The data are shown as the mean ± SD.

The identification of proteins that showed increased binding to PSRC1 according to non‐labelled quantitative proteomics revealed that after transfection of RAW264.7 cells with Ad‐PSRC1, thermograms and volcano plots suggested that ANXA2 binding was markedly elevated in the Ad‐PSRC1 group compared to Ad‐GFP in response to ox‐LDL stimulation (Figure 1A,B). Representative peptides of the ANXA2 protein identified by mass spectrometry are shown in Figure S1A,B. Immunofluorescence staining of RAW264.7 cells revealed the colocalization of PSRC1 with ANXA2 in macrophages (Figure 1C,D). Co‐IP experiments confirmed that PSRC1 interacts with ANXA2 in Raw264.7 cells and that binding is significantly increased after ox‐LDL stimulation (Figure 1E). ANXA2 levels in cell culture supernatants were assessed using ELISA, and the levels were significantly increased in cultures treated with ox‐LDL (*p* < .01, Figure 1F). When PSRC1 was overexpressed, the levels of ANXA2 in the cell culture supernatants were decreased in Ad‐PSRC1 group compared to Ad‐GFP (*p* < .01, Figure 1G). These outcomes indicate that PSRC1 increased the binding of ANXA2 and reduced ANXA2 secretion in the context of ox‐LDL stimulation.

### 3.2 ANXA2 promote AS progression in vivo

**FIGURE 2:**
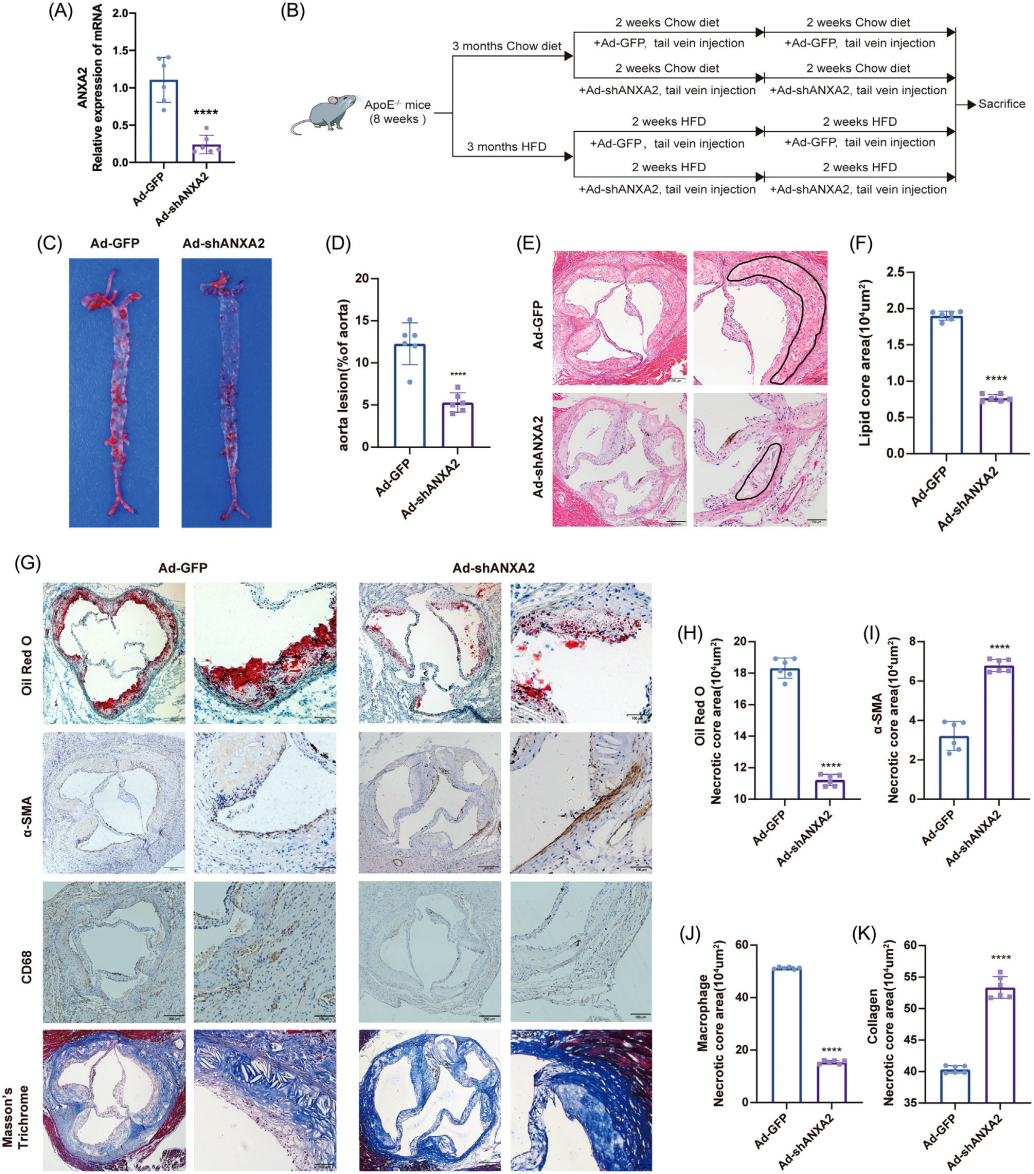
ANXA2 promotes AS progression in vivo. (A) Experimental timeline. (B and C) Oil Red O staining of ApoE^−/−^ mouse aortic bulk plaques after injection with Ad‐green fluorescent protein (GFP) and Ad‐shANXA2, 12.28% ± 2.48% versus 5.29% ± 1.14%, *n* = 6 per group, Student's *t* test, ****p < .0001. (D and E) HE staining of aortic root sections of the mice in the Ad‐GFP and Ad‐shANXA2 groups shows the lipid core area, 1.91 ± .06 versus .77 ± .05(10^4^ μm^2^). Left: scale bar = 100 μm. Right: scale bar = 200 μm. *n* = 6 per group, Student's *t* test, ****p < .0001. (F–J) Cross‐sections of the aortic roots of ApoE^−/−^ mice injected with Ad‐GFP or Ad‐shANXA2 were stained with Oil Red O to identify lipids (18.32 ± .64 vs. 11.23 ± .35 [10^4^ μm^2^]), α‐SMA to identify smooth muscle cells (SMCs) [3.22 ± .73 vs. 6.79 ± .32(10^4^ μm^2^)], CD68 to identify macrophages (51.25 ± .42 versus 15.43±.68 [10^4^ μm^2^]), and Masson's trichrome to identify collagen (40.35 ± .57 vs. 53.35 ± 1.72 [10^4^ μm^2^]). Left: scale bar = 100 μm. Right: scale bar = 200 μm. *n* = 6 per group, Student's *t* test, ****p < .0001. The data are shown as the mean ± SD.

To investigate the effect of ANXA2 on AS progression, we first constructed an Ad‐shANXA2 adenoviral vector and injected mice with this vector via the tail vein (Figure 2A). The experimental timeline is shown in Figure 2B. In the chow diet groups, there were no obvious atherosclerotic plaques (Figure S2A,B). There was no discernible difference in body weight between the two groups following high‐fat diet feeding (Figure S2C). The aorta macroscopic Oil Red O staining results revealed that the plaque area in the Ad‐shANXA2 group was dramatically decreased when compared to Ad‐GFP (*p* < .05, Figure 2C, D), and aortic root HE staining suggested a reduction in the aortic root plaque area in the Ad‐shANXA2 (*p* < .05, Figure 2E, F). This suggests that knockdown of ANXA2 delayed the formation of AS plaques. Regarding lipid levels, TG, CHOL and LDL were significantly lower in the Ad‐shANXA2 than in the control, and HDL was significantly higher in the Ad‐shANXA2 than in the control (Figure S2D–G).

The results of TG and collagen staining revealed that compared with the control group, the TG in the aortic roots of the Ad‐shANXA2 mice were decreased (*p* < .0001), and the aortic root fibre areas were increased (*p* < .0001). The results of α‐SMA suggested that the areas of SMCs in the aortic roots of the Ad‐shANXA2 group were increased compared with the control group (*p* < .0001). The CD68 immunohistochemical staining results revealed that the areas of macrophage infiltration in the aortic roots of the Ad‐shANXA2 mice were decreased compared with those of the control mice (*p* < .0001, Figure 2G,K). These results suggested that ANXA2 knockdown increased aortic root plaque stability.

Serum was taken from each group of mice, and its ANXA2 and inflammatory factor concentrations were assessed. According to the findings, the levels of ANXA2 were significantly lower following the injection of Ad‐shANXA2 (Figure S3A), and the levels of IL‐1β and IL‐6 in serum were also significantly reduced (Figure S3B,C), suggesting that ANXA2 can also affect the inflammatory response in vivo.

### 3.3 PSRC1 attenuates AS progression via ANXA2

**FIGURE 3:**
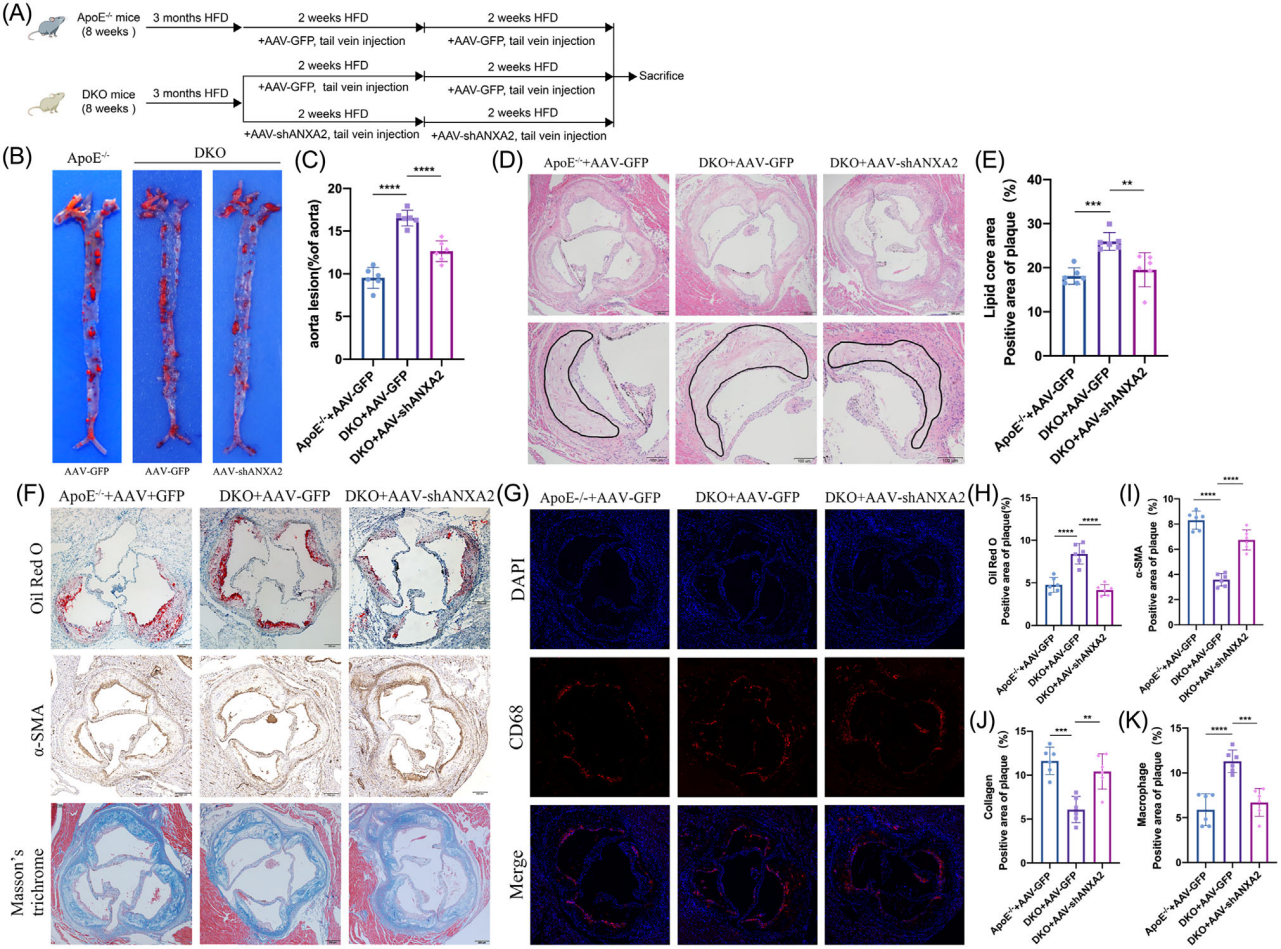
PSRC1 attenuates AS progression via ANXA2. (A) Experimental timeline. (B and C) Oil Red O staining of aortic bulk plaques from the three groups, 9.52%±1.23% versus 16.52%±.92% **versus** 12.64%±1.22%, *n* = 6 per group, one‐way ANOVA, ****p < .0001. (D and E) HE staining of aortic root sections of mice in the three groups showing the lipid core area, 18.11%±1.86% versus 25.99%±2.04% versus 19.57%±3.87%. Upper: scale bar = 100 μm. Bottom: scale bar = 200 μm, *n* = 6 per group, one‐way ANOVA, **p < .01, ***p < .001. (F–K) Cross‐sections of the aortic root were stained with Oil Red O for lipids(4.77%±.87% vs. 8.41%±1.21% vs. 4.17%±.64%), α‐SMA for smooth muscle cells (SMCs) (8.31%±.72% vs. 3.58%±.50% vs. 6.74%±.80%), Masson's and trichrome for collagen (11.62%±1.56% vs. 6.08%±1.48% vs. 10.42%±2.01%), and CD68 for macrophages(5.90%±1.77% vs. 11.3%±1.26% vs. 6.71%±1.56%) in the three groups. Scale bar = 100 μm, *n* = 6 per group, one‐way ANOVA, **p < .01, ***p < .001, ****p < .0001. The data are shown as the mean ± SD.

To investigate whether PSRC1 acts through ANXA2, we first constructed PSRC1^−/−^ ApoE^−/−^ (DKO) mice by CRISPR‒Cas9‐mediated knockdown of the PSRC1 gene in ApoE^−/−^ mice (Figure S3A–D) and used AAV‐shANXA2 to knock down ANXA2 in macrophages (Figure S5A,B). The experimental timeline is shown in Figure 3A. Immunofluorescence staining of cross‐sections of the mouse atherosclerotic aortic roots identified GFP in the F4/80+ area and the expression and distribution of PSRC1 and ANXA2 within the plaque (Figure S5C,D). There was no discernible change in body weight among the three groups (Figure S6A). In the DKO+AAV‐GFP group, TG, CHOL and LDL were significantly higher than ApoE^−/−^+AAV‐GFP, and HDL was significantly lower than ApoE^−/−^+AAV‐GFP, with no significant differences between DKO+AAV‐GFP and DKO+AAV‐shANXA2 (Figure S6B–E). Oil Red O staining of the aortic bulk and aortic root suggested that the DKO+AAV‐GFP group had significantly more plaques than the ApoE^−/−^+AAV‐GFP group (*p* < .0001 and *p* < .0001, Figure 3B,C,F,H). When ANXA2 was knocked down in the macrophages of DKO mice, the plaque area was markedly reduced (*p* < .0001 and *p* < .0001, Figure 3B,C,F,H).

The results of HE staining and Masson's trichrome staining of the aortic roots in the three groups of mice showed that the areas of the lipid core in the aortic roots of the DKO mice were increased compared with those of the ApoE^−/−^ mice (*p* < .001, Figure 3D–E) and that the areas of aortic root fibres were decreased (*p* < .001, Figure 3F,J). α‐SMA immunohistochemical staining suggested that the areas of SMCs in the aortic roots of the DKO mice were smaller than those of the ApoE^−/−^ mice (*p* < .0001, Figure 3F,I). CD68 immunofluorescence staining revealed an increase in the areas of macrophage infiltration in the aortic roots of the DKO mice compared to those of the ApoE^−/−^ mice (*p* < .0001, Figure 3G,K). These results suggest that aortic root plaque stability is significantly reduced when PSRC1 is knocked out. After ANXA2 knockdown in DKO mouse macrophages, the aortic root lipid core areas were decreased (*p* < .01, Figure 3D–E), the fibre areas were increased (*p* < .01, Figure 3F,J), the smooth muscle cell areas were increased (*p* < .0001, Figure 3F,3I) and the macrophage infiltration area was decreased (*p* < .001, Figure 3G,3K). All of these results suggest that ANXA2 knockdown in DKO mouse macrophages increases plaque stability.

The levels of ANXA2 and inflammatory factors in serum were measured by ELISA. The findings showed that ANXA2 levels were substantially higher in DKO mice compared to ApoE^−/−^ mice and significantly reduced after the injection of AAV‐shANXA2 (Figure S7A). Similarly, the levels of IL‐1β and IL‐6 in serum were significantly increased in DKO mice and were reduced after ANXA2 knockdown in DKO mouse macrophages (Figure S7B,C), suggesting that ANXA2 can also affect the inflammatory response in vivo.

### 3.4 ANXA2 affects inflammation by promoting STAT3 phosphorylation

**FIGURE 4:**
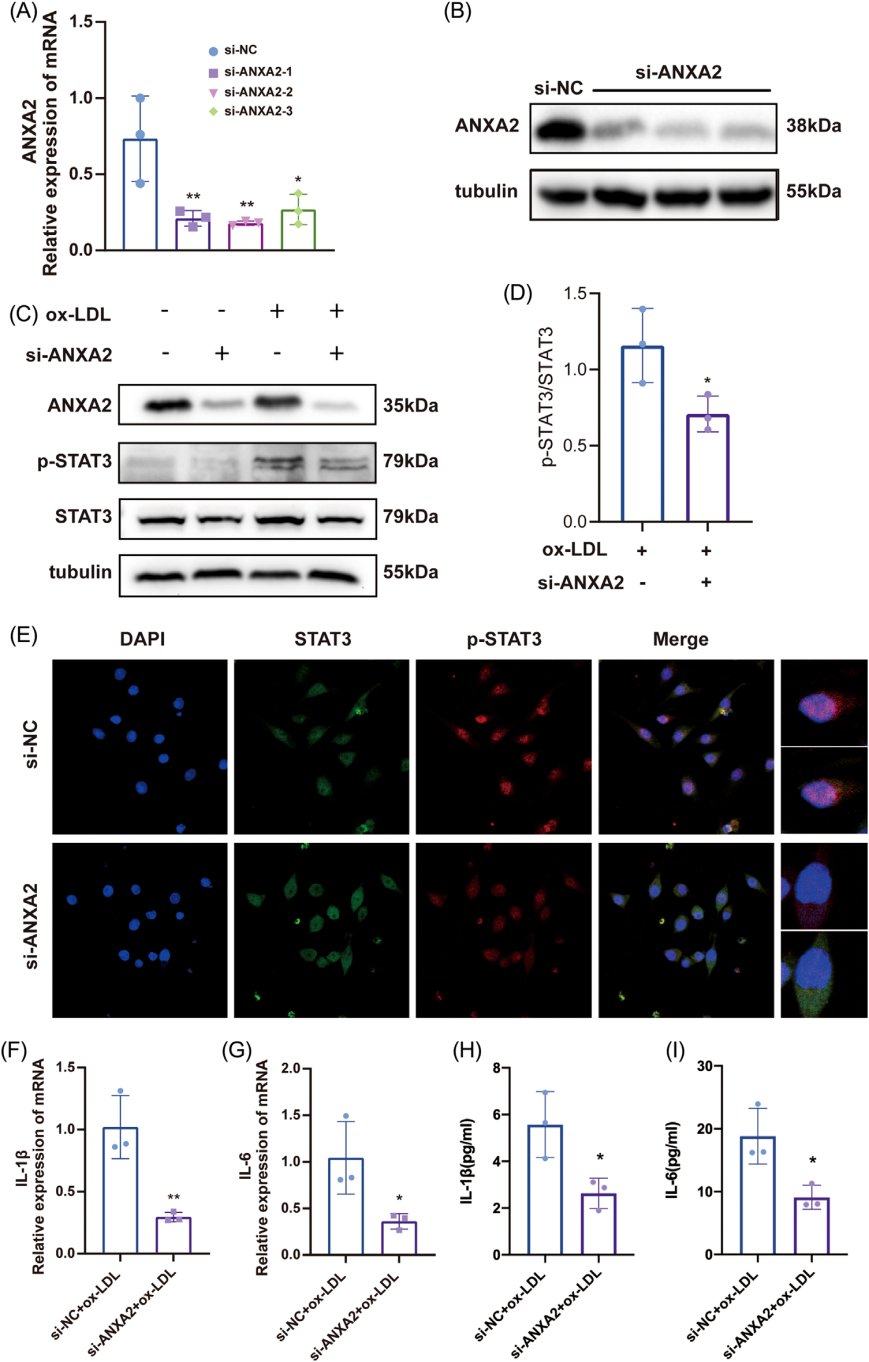
ANXA2 affects inflammation by promoting STAT3 phosphorylation. Raw264.7 cells were transfected with si‐NC and si‐ANXA2 for 30 h. RNA and protein were extracted for RT‐qPCR (A) and Western blotting (B) to verify transfection efficiency, respectively. *n* = 3 per group, Student's *t* test, *p < .05, **p < .01. Raw264.7 cells transfected with si‐NC and si‐ANXA2 were treated with oxidized‐low‐density lipoprotein (ox‐LDL) for 24 h. Western blotting was performed to measure p‐STAT3 and STAT3 expression levels (C) and p‐STAT3/STAT3 was quantified (D), *n* = 3 per group, Student's *t* test, *p < .05. (E) Immunofluorescence staining showed the changes in the localization and content of p‐STAT3 in Raw264.7 cells after transfection with si‐NC and si‐ANXA2. IL‐1β (F), IL‐6 (G)mRNA levels and IL‐1β (H), IL‐6 (I) concentrations in cell culture supernatants were measured after transfection with si‐NC and si‐ANXA2 and treatment with ox‐LDL for 24 h, *n* = 3 per group, Student's *t* test, *p < .05, **p < .01.

To further investigate the specific mechanism by which ANXA2 affects the inflammatory response, we found that ANXA2 is able to stimulate tumour cell proliferation by altering STAT3 phosphorylation levels.^14^ After we constructed small interfering RNA to target ANXA2 and verified the knockdown efficiency at the transcription and protein levels (Figure 4A,B), si‐NC and si‐ANXA2 were transfected into Raw264.7 cells. The results showed that the reduced expression level of ANXA2 was followed by an equally significant reduction in the p‐STAT3 levels (*p* < .05, Figure 4C,D). We also detected ERK1/2 and P38 in the MAPK pathway and showed that the phosphorylation of ERK1/2 and P38 was unaffected by ANXA2 knockdown (Figure S8). The immunofluorescence staining results showed that knockdown of ANXA2 expression reduced the p‐STAT3 expression level in the nucleus (Figure 4E). Moreover, the mRNA levels and secretion of inflammatory factors into cell culture supernatants were considerably lower in the si‐ANXA2 group compared with si‐NC (Figure 4F–I). In parallel, we verified the same results by ANXA2 and p‐STAT3 immunofluorescence staining in cross‐sections of AAV‐GFP and AAV‐shANXA2 mouse aortic roots (Figure S9). Therefore, we suggest that ANXA2 can influence the inflammatory response by affecting STAT3 phosphorylation and its translocation into the nucleus. In addition, since altered lipid levels were found in animals after knockdown of ANXA2, we examined mRNA levels of lipid metabolism‐related proteins after transfection with si‐ANXA2 in Raw2647 and AML12 cells, respectively, and found no significant differences (Figure S10), and the mechanism by which ANXA2 affects lipid levels needs to be further explored.

### 3.5 The transcription factor SP1 inhibitor Mith elevates PSRC1 expression in macrophages

**FIGURE 5:**
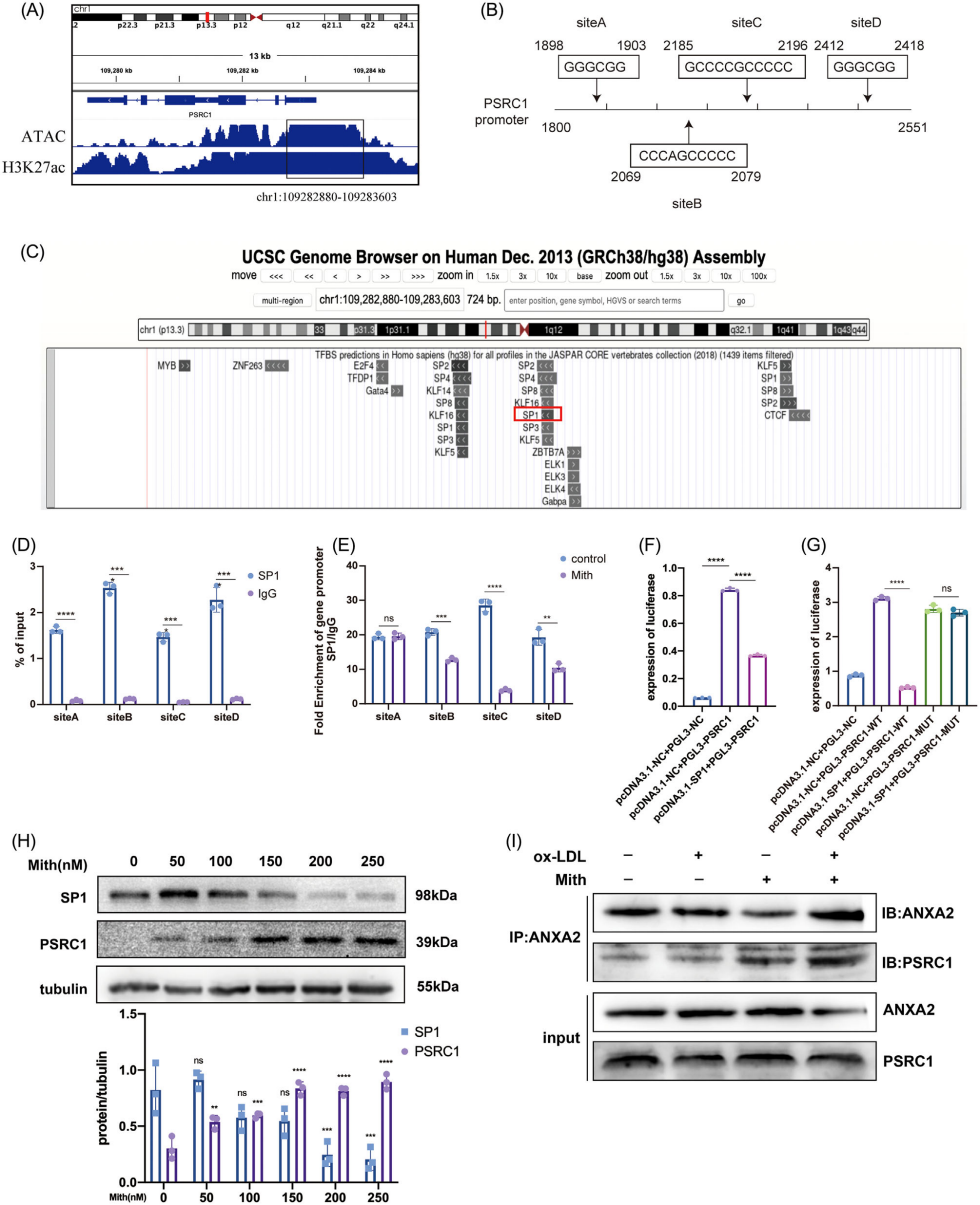
The transcription factor SP1 inhibitor Mith elevates PSRC1 expression in macrophages. (A) THP‐1 cells were subjected to ATAC‐seq and ChIP‐seq with an anti‐H3K27ac antibody, and the human PSRC1 gene open region locus was determined (i.e., promoter region). (B) Schematic representation of the possible binding sites of SP1 to the PSRC1 promoter. (C) UCSC was used to predict transcription factors that may bind to the PSRC1 promoter. (D) THP‐1 cells were used for ChIP. ChIP DNA was isolated via SP1 antibody or IgG immunoprecipitation, and it was used to perform RT‐qPCR with PSRC1 promoter‐specific primers. n = 3 per group, Student's *t* test, ****p < .0001. (E) ChIP was performed separately for THP‐1 cells after 24 h of treatment with 200 nM Mith or PBS. ChIP DNA was immunoprecipitated with an anti‐SP1 antibody, followed by RT‒qPCR with PSRC1 promoter‐specific primers. *n* = 3 per group, Student's *t* test, **p < .01, ***p < .001, ****p < .0001. After transfection of THP‐1 cells with the SP1 plasmid (F) and PSRC1 promoter region mutant plasmid (G), the PSRC1 promoter luciferase activity was measured by a dual‐luciferase reporter system. *n* = 3 per group, one‐way ANOVA, ****p < .0001. (H) Representative Western blot and of THP‐1 cells treated with different concentrations of Mith. *n* = 3 per group, Student's *t* test, each group compared with the 0nM group, **p < .01, ***p < .001, ****p < .0001. (I) THP‐1 cells were treated with 100 μg/mL oxidized‐low‐density lipoprotein (ox‐LDL) or 200 nM Mith and then immunoprecipitated using an anti‐ANXA2 antibody. The interacting proteins were detected by Western blotting. +: positive control; −: negative control.

In our previous study, PSRC1 overexpression was achieved by viral vectors; however, the safety of this method when applied directly to patients in the clinic is unclear, so we aimed to identify a drug that could be used instead of viral vectors to increase PSRC1 expression levels. We performed ATAC‐seq and ChIP‐seq on THP‐1 cells and found that there is an open region in the PSRC1 initiation region, which is the promoter region (Figure 5A). Then, we predicted the transcription factors that possibly bind to this region by UCSC; we found that SP1 was more likely to bind to the PSRC1 promoter region (Figure 5C). The SP/KLF family of transcription factors includes SP1 as a member. Due to the presence of other SPs in the predicted results, we examined PSRC1 mRNA levels by siRNA inhibition of SP2, SP3, SP4 and SP8 expression, and the results showed no significant difference in PSRC1 levels (Figure S11A–D). A search revealed a non‐specific inhibitor of SP1, Mith, which acts mainly by inhibiting the binding of SP1 to the GCbox in the downstream promoter region and by promoting protein degradation.^15^ We subsequently verified whether SP1 plays a role in regulating PSRC1 expression. First, we predicted four possible binding sites of SP1 to PSRC1 (Figure 5B), and ChIP‒qPCR experiments revealed that SP1 binds to all four sites (Figure 5D). In addition, after Mith treatment, the binding of SP1 to sites B, C and D in the PSRC1 promoter region was considerably reduced compared to that of the control, while no significant change was observed at site A (Figure 5E). Wild‐type and mutant PSRC1 vectors were constructed, and dual luciferase experiments were performed. The results showed that SP1 could inhibit PSRC1 promoter luciferase activity, and this inhibitory effect disappeared after the promoter region was mutated (Figure 5F,G). These experimental results indicate that SP1 interacts with the PSRC1 promoter region and exerts an inhibitory effect.

To visually demonstrate the regulatory effect of SP1 on PSRC1, we treated THP‐1 cells with Mith concentration gradients, and the Western blotting results showed that PSRC1 expression gradually increased with increasing Mith concentration (Figure 5H). Co‐IP experiments showed that Mith increased the binding of PSRC1 to ANXA2 in THP‐1 cells (Figure 5I). Mith attenuated the expression of IL‐1β, IL‐6 and TNF‐α in macrophages but did not affect the expression of TGF‐β, IL‐4 and IL‐10. After Mith intervention, IL‐1, IL‐6 and ANXA2 levels in cell culture supernatants were markedly reduced (Figure S12A–I). In addition, Mith was able to elevate LXR mRNA levels but did not affect ABCA1, ABCG1, SRB1 and SREBP2 in macrophages and PCSK9 expression in hepatocytes (Figure S13).

### 3.6 The SP1 inhibitor Mith attenuates AS progression via PSRC1

**FIGURE 6:**
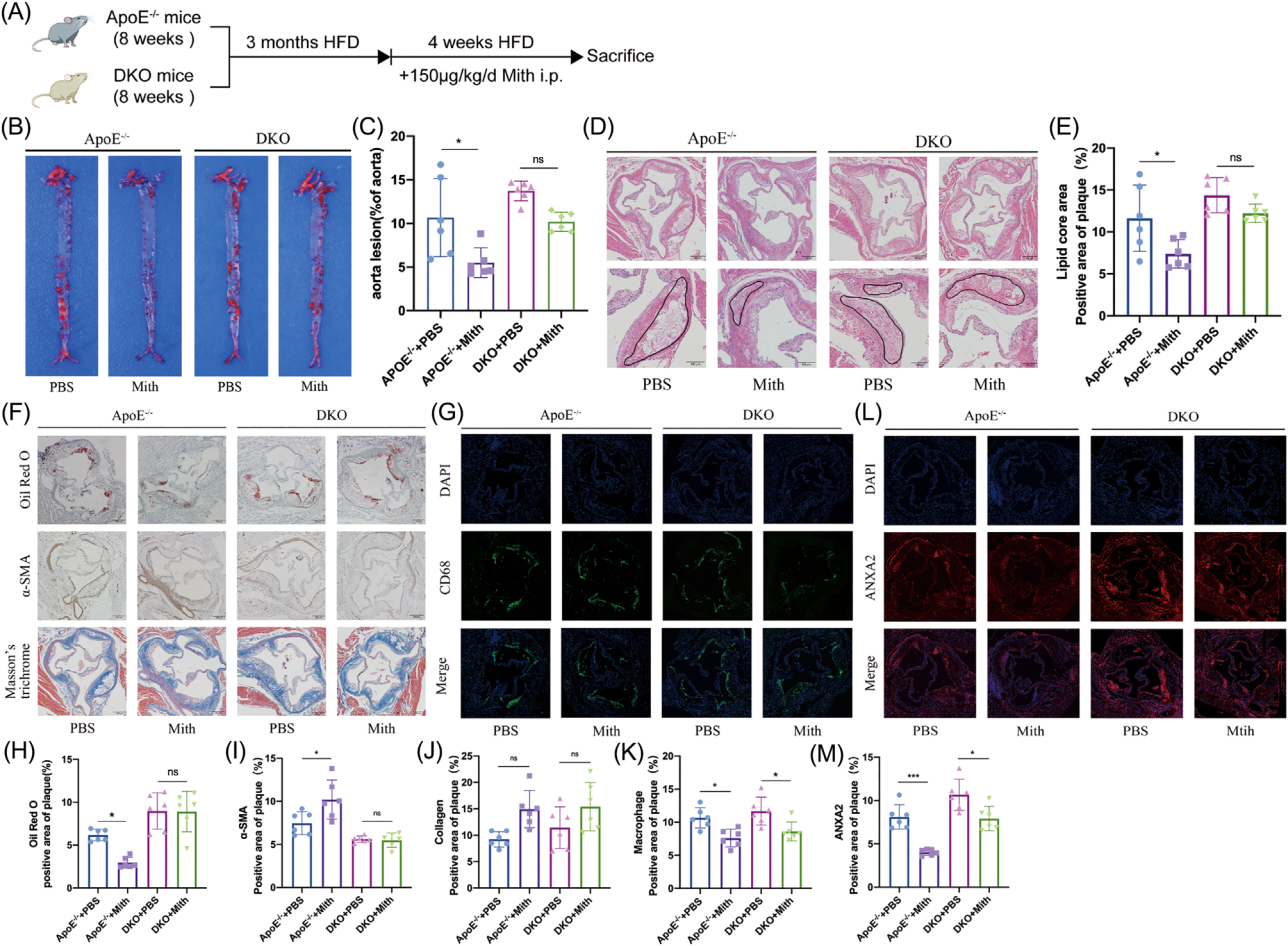
The SP1 inhibitor Mith attenuates AS progression via PSRC1. (A) Experimental timeline. (B and C) Oil Red O staining of ApoE^−/−^ mouse and DKO mouse aortic bulk plaques after intraperitoneal injection of Mith or control, 10.66% ± 4.47% versus 5.50% ± 1.71% versus 13.72% ± 1.14% versus 10.18% ± 1.09%, *n* = 6 per group, one‐way ANOVA, *p < .05. (D and E) HE staining of aortic root sections of mice in the four groups showing the lipid core area, 11.63% ± 3.95% versus 7.39% ± 1.70% versus 14.38% ± 2.09% versus 12.23% ± 1.09%. Left: scale bar = 100 μm. Right: scale bar = 200 μm. *n* = 6 per group, one‐way ANOVA, *p < .05. (F–K) Cross‐sections of aortic roots from the four groups were stained with Oil Red O to identify lipids (6.17% ± .67% vs. 2.95% ± .63% vs. 8.98% ± 2.12% vs. 8.92% ± 2.35%), α‐SMA to identify SMCs (7.46% ± 1.32% vs. 10.21% ± 2.26% vs. 5.61% ± .36% vs. 5.48% ± .83%), Masson's trichrome to identify collagen(9.23% ± 1.44% vs. 14.94% ± 3.51% vs. 11.44% ± 3.93% vs. 15.4% ± 4.58%), and CD68 to identify macrophages(10.66% ± 1.53% vs. 7.61% ± 1.33% vs. 11.68% ± 2.10% vs. 8.61% ± 1.43%); scale bar = 100 μm. *n* = 6 per group, one‐way ANOVA, *p < .05. (L and M) Immunofluorescence showing the area of ANXA2 in the aortic root sections of the four groups, 8.11% ± 1.41% versus 4.03% ± .35% versus 10.67% ± 1.80% versus 7.92% ± 1.40%, scale bar = 100 μm. *n* = 6 per group, one‐way ANOVA, *p < .05, ***p < .001. The data are shown as the mean ± SD.

**FIGURE 7:**
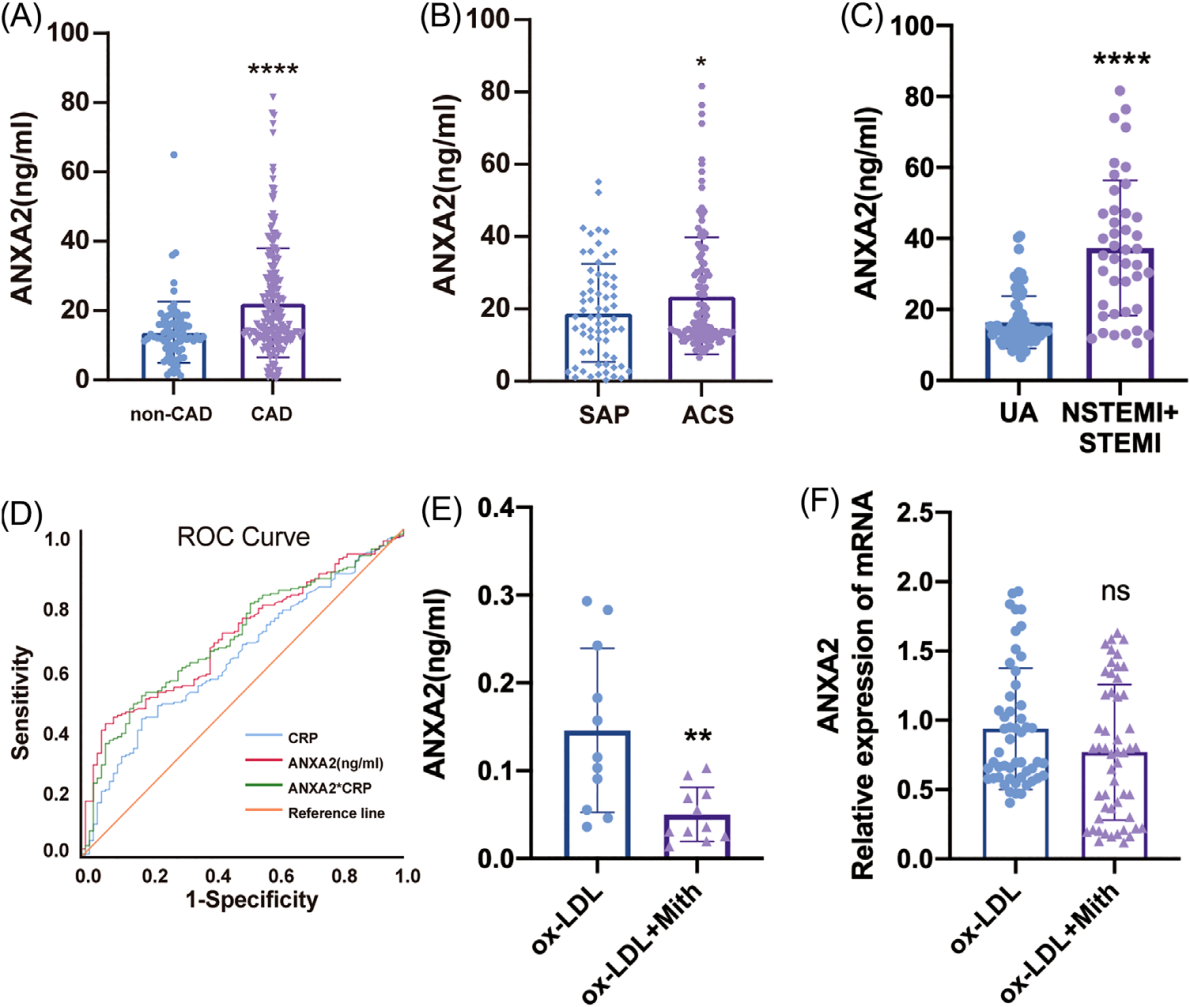
Correlation of ANXA2 with CAD. (A) ANXA2 levels in serum of non‐CAD (*n* = 80) and CAD (*n* = 200) patients were assessed by ELISA, 13.79 ± 8.79 versus 22.21 ± 15.71 (ng/mL), Student's *t* test, ****p < .0001. (B) CAD patients were divided into SAP (*n* = 66) and ACS (*n* = 119) groups, and serum ANXA2 levels were measured by ELISA in both groups. 18.94 ± 13.54 versus 23.65 ± 16.17 (ng/mL), Student's *t* test, *p < .05. (C) ACS patients were divided into UA (*n* = 77) and NSTEMI+STEMI (*n* = 42), and serum ANXA2 levels were measured by ELISA in both groups. 16.38 ± 7.38 versus 37.33 ± 19.02 (ng/mL), Student's *t* test, *p < .05. (D) CRP, ANXA2 and ANXA2*CRP predict the ROC curve of CAD. (E) Peripheral blood monocytes from CAD patients were divided into two groups and stimulated with PBS (*n* = 11) or 200 nM Mith (*n* = 11) for 24 h followed by 100 μg oxidized‐low‐density lipoprotein (ox‐LDL) for 24h, and ANXA2 levels in cell culture supernatants were detected by ELISA. Student's *t* test, **p < .01, ****p < .0001. (F) Peripheral blood monocytes from CAD patients were divided into two groups and stimulated with PBS (*n* = 52) or 200 nM Mith (*n* = 52) for 24 h followed by 100 ug/mL ox‐LDL for 24 h, and ANXA2 mRNA in monocytes was detected by RT‐qPCR. Student's *t* test, ns *p* > 0.05.

In the above experiments, we verified that Mith increases PSRC1 expression levels in macrophages in vitro. Combined with the conclusion from previous work that PSRC1 delays AS progression in ApoE^−/−^ mice, we conducted another study to further verify the interrelationship between Mith and PSRC1 by feeding ApoE^−/−^ mice and DKO mice a HFD for 3 months. The experimental timeline is shown in Figure 7A. The average body weights of the four groups were nearly identical (Figure S14A). The DKO group greatly outperformed the ApoE^−/−^ group in terms of blood lipids, although Mith injection had no statistically significant impact on lipid levels (Figure S14B–E). When compared to the control group, we discovered that the Mith group had less aortic bulk plaque and aortic root plaque (*p* < .05 and *p* < .05, Figure 6B–E). However, there was no discernible difference between the Mith and the control in the DKO mice (*p* = .1 and *p* = .4, Figure 6B–E).

The results of TG and collagen staining of the aortic roots of the four groups of mice described above suggested that in ApoE^−/−^ mice, the areas of TG in the aortic roots were decreased (*p* < .05, Figure 6F,H) and the areas of fibres in the aortic roots were increased (*p* = .053, Figure 6F,J) in the Mith group compared with the control group. The areas of the TG in the aortic roots were decreased (*p* = .9, Figure 6F,H), and the areas of aortic root fibres were increased (*p* = .25, Figure 6F,J) compared with those of the control group of DKO mice. α‐SMA immunohistochemical staining showed that while no discernible difference was seen between the Mith group and the control group of DKO mice (*p* = .9, Figure 6F,I), regions of aortic root SMCs were increased in the Mith group as compared to the control group of ApoE^−/−^ mice (*p* < .05, Figure 6F,I). In the Mith group compared to the control of ApoE^−/−^ mice (*p* < .05, Figure 6G,K), as well as in the Mith group compared to the control of DKO mice (*p* < .05, Figure 6G,K), CD68 immunofluorescence labelling revealed a decrease in the aortic root macrophage regions. Mith significantly reduced ANXA2 secretion in ApoE^−/−^ mice compared to the control (*p* < .05, Figure 6L,M), according to ANXA2 immunofluorescence staining, and Mith also reduced ANXA2 secretion in DKO mice (*p* < .05, Figure 6L,M), but slightly less than in ApoE^−/−^ mice. Serum ANXA2 levels exhibited the same differences as those within the aortic root plaques (Figure S14F). Regarding serum inflammatory factor levels, DKO mice had significantly higher levels of proinflammatory factors than ApoE^−/−^ mice, and after injection of Mith, the levels of IL‐1β and IL‐6 in ApoE^−/−^ mice were significantly reduced but not in DKO mice (Figure S14G,H).

These results indicated that injection of Mith into ApoE^−/−^ mice delayed AS plaque formation and increased plaque stability, and knockdown of PSRC1 also delayed AS progression to some extent but to a lesser extent than in ApoE^−/−^ mice.

### 3.7 The relevance of the ANXA2 level to CAD

Peripheral blood specimens from 80 non‐CAD patients and 200 CAD patients were collected, and Table 1 shows the baseline data. The serum ANXA2 levels were measured by ELISA after serum separation, and the results suggested that the patients with CAD had considerably higher serum ANXA2 levels than those without CAD (*p* < .0001, Figure 7A). Further subgroup analysis was performed, and the CAD patients were divided into acute coronary syndrome (ACS) and stable angina pectoris (SAP) groups (baseline data are shown in Table 2). The ANXA2 levels were elevated in ACS patients compared to SAP patients (*p* < .05, Figure 7B). ACS patients were further divided into UA, NSTEMI+STEMI, and it was found that ANXA2 levels were significantly lower in UA patients than in NSTEMI and STEMI (Figure 7C). In our included patients, single‐factor logistic regression analysis identified ANXA2, male sex, diabetes mellitus, smoking, blood creatinine, and leukocytes as risk factors for developing coronary heart disease. After adjustment for confounders, multivariable logistic regression analysis found that ANXA2, male sex, diabetes, leukocytes, and blood creatinine were independent risk factors for CAD (Table S1). In CAD patients, the predictive value of ANXA2, CRP, and ANXA2 combined with CRP for CAD was analysed using ROC curves, and the results showed that ANXA2 had the largest area under the curve (Figure 7D, the area under the ROC curve is shown in Table S2). These findings imply that ANXA2 might predict the development of CAD.

**TABLE 1:** Baseline and clinical characteristics of study population.

	Total	non‐CAD	CAD	*p*‐Value
**Sex (male)**	280 (199)	80 (43)	200 (156)	.000053*
**Ages (years)**	60 (53‐66)	59.22 ± 9.62	59.36 ± 8.44	.937
**BMI (kg/m^2^)**	24.65 (22.82‐26.70)	24.71 ± 2.67	25.00 ± 3.17	.507
**Smoke (%)**	126 (45.49)	25 (31.6)	101 (51.0)	.003*
**Hypertension (%)**	150 (54.15)	38 (48.10)	112 (56.57)	.202
**diabetes mellitus (%)**	82 (29.60)	14 (17.72)	69 (34.34)	.006*
**Leukocyte (×10^9^/L)**	7.25 (5.95–9.13)	6.41 (5.54–7.83)	7.53 (6.39–9.36)	.000096*
**CRP**	1.95 (.82–4.55)	1.39 (.63–2.53)	2.16 (.90–6.24)	.004*
**Creatinine (mol/L)**	82 (69‐98)	75 (64–89)	86 (72.75–101.25)	.001*
**Cholesterol (mmol/L)**	4.60 ± 1.56	4.15 (3.65–5.04)	4.71 (3.80–5.38)	.04*
**Triglycerides (mmol/L)**	1.49 (1.02–2.36)	1.45 (1.01–2.22)	1.52 (1.04–2.43)	.568
**HDL‐C (mmol/L)**	1.05 (.89–1.28)	1.14 (.96–1.43)	1.03 (.87–1.22)	.14
**LDL‐C (mmol/L)**	2.80 ± 1.22	2.62 ± .97	2.87 ± 1.15	.088
**ANXA2 (ng/mL)**	15.01 (11.36–24.78)	12.91 (9.08–16.52)	16.53 (12.18–29.32)	.000007*

Abbreviations: CRP, C‐reative protein; HDL‐C, high density lipoprotein‐cholesterol; LDL‐C, low density lipoprotein‐cholesterol.

* *p* < .05.

**TABLE 2:** Baseline and clinical characteristics of SAP and ACS patients.

	SAP	ACS	*p*‐Value
**Sex (male)**	66 (54)	119 (91)	.397
**Ages (years)**	61.49 ± 7.31	58.41 ± 8.53	.005*
**BMI (kg/m^2^)**	25.28 ± 3.02	24.79 ± 3.18	.284
**Smoke (%)**	35 (53.03)	62 (52.10)	.903
**Hypertension (%)**	43 (65.15)	64 (53.78)	.134
**Diabetes mellitus (%)**	27 (40.90)	40 (33.61)	.323
**Leukocyte (×10^9^/L)**	7.43 ± 1.90	8.65 ± 2.89	.001*
**CRP**	1.41 (.61–4.1)	3.17 (1.13–8.07)	.001*
**Creatinine (mol/L)**	83 (72.5–106.5)	86.5 (73–99.75)	.005*
**Cholesterol (mmol/L)**	4.50 ± 1.19	4.76 ± 1.32	.115
**Triglycerides (mmol/L)**	1.40 (1.01–1.94)	1.58 (1.02–2.47)	.488
**HDL‐C (mmol/L)**	1.01 (.87–1.16)	1.02 (.87–1.24)	.061
**LDL‐C (mmol/L)**	2.76 ± 1.12	2.92 ± 1.18	.265
**ANXA2 (ng/mL)**	18.87 ± 14.01	23.42 ± 15.99	.045*

Abbreviations: CRP, C‐reative protein; HDL‐C, high density lipoprotein‐cholesterol; LDL‐C, low density lipoprotein‐cholesterol.

* *p* < .05

To investigate the effect of Mith on monocytes from CAD patients, peripheral blood monocytes from the CAD patients described above were isolated and divided into the control and Mith groups. The results suggested that the level of ANXA2 in the monocyte cell culture supernatants from CAD patients was significantly reduced after treatment with Mith compared with the control (*p* < .01, Figure 7E). There was no marked difference in ANXA2 mRNA levels after Mith treatment of monocytes from CAD patients (Figure 7F). To further validate the results, we stimulated THP‐1 with 100ug/mL ox‐LDL and performed the same experiments, which suggested that ANXA2 release from THP‐1 was reduced after Mith stimulation, while mRNA levels were no discernible differences between the two groups, with the same results as in peripheral blood monocytes (Figure S15).

## 4 DISCUSSION

ASCVD is currently one of the major life‐threatening diseases in the world, and AS is the main pathological basis of the disease. In addition to abnormal lipid levels, inflammation is also one of the major pathological mechanisms underlying ASCVD.^16^ Based on previous studies, we found that in inflammatory macrophages, PSRC1 binds to ANXA2 and reduces its extracellular release, thus inhibiting its proinflammatory effects; however, ANXA2 was able to promote inflammation in macrophages, thereby accelerating the progression of AS. We found that SP1 is an upstream inhibitory transcription factor of PSRC1, and the SP1 inhibitor Mith can increase PSRC1 expression in macrophages. These findings imply that PSRC1/ANXA2 may be a prospect target for the clinical therapeutic of ASCVD, and Mith is also expected to be an effective drug for ASCVD treatment.

In the development of AS, macrophages, which are the main cells involved in both lipid metabolism and inflammatory reactions of the body, can absorb a large amount of cholesterol and other lipids in blood circulation; the deposition of these lipids in macrophages causes their gradual differentiation into foam cells, ultimately resulting in the formation of atherosclerotic plaques. On the other hand, macrophages secrete a variety of inflammatory factors and initiate inflammatory reactions, and large numbers of macrophages and T lymphocytes accumulate in the peripheral zone, fibrous cap and core of AS plaques. As the disease develops, the number of macrophages gradually increases from the lipid core to the surface layer of the fibrous cap, affecting the physiological function of the vascular system as well as the progression and stability of the plaque.^6^ It has been shown that the levels of inflammatory lymphocytes in peripheral blood remain high in patients with acute myocardial infarction in whom LDL‐C is controlled at lower levels.^17^ The CANTOS study found that in enrolled patients with myocardial infarction with adequate lipid control, the use of a monoclonal antibody that targets IL‐1β and canakinumab further reduced the incidence of major cardiovascular events.^5^ This suggests that anti‐inflammatory therapy is an effective means of treating AS.

In a prior study, we demonstrated the role of PSRC1 in regulating lipid metabolism, reducing the inflammatory response and thus influencing the onset of AS.^12^ As a microtubule‐binding protein, PSRC1 can bind to microtubule proteins, which play roles in cellular support, to maintain microtubule stability at one end and to other intracellular proteins to exert biological effects at the other end. However, PSRC1 is present only in the cell cytoplasm, and it is not observed in the cell membrane or outside the cell. The specific mechanism by which PSRC1 affects the release of inflammatory factors and transmits intracellular signals outside of the cell needs to be further explored. A recent study revealed that PSRC1 can attenuate AS progression by regulating the intestinal flora and the pathway related to its metabolite trimethylamine‐N‐oxide (TMAO), that PSRC1 can inhibit TMAO‐induced cholesterol aggregation and the inflammatory response and that increased TMAO levels can also inhibit PSRC1 expression and promote AS.^18^ We found through proteomic studies that PSRC1 binds to ANXA2 in AS. The significant increase in PSRC1 binding in AS suggests that PSRC1 exerts anti‐AS effects through ANXA2. ANXA2 plays different roles in different cells and in different diseases. Studies have shown that ANXA2 on cell membranes is able to act as a receptor that binds to fibrinogen and allows it to bind to tissue plasminogen activator, promoting fibrin formation.^19^, ^20^ In tumours, ANXA2 is able to promote tumour cell migration and neovascularization.^19^ The role played by ANXA2 in AS has not been elucidated; one study found that in the liver cell cytosol, ANXA2 is able to bind to the proprotein convertase subtilis protease/kexin type 9 (PCSK9) and inhibit the degradation of LDL receptor by PCSK9.^21^, ^22^ However, it has also been found that ANXA2 mediates the release of metalloproteinase‐9 (MMP‐9), and statins reduce the release of MMP‐9 by inhibiting ANXA2 expression and stabilizing plaques.^23^ It has also been found that ANXA2 mediates the migration of macrophages to AS plaques and increases the release of inflammatory factors, promoting an inflammatory response.^24^ Moreover, extracellular ANXA2 was shown to bind to TLR4 receptors on the surface of macrophages, promoting the release of IL‐1β, IL‐6 and TNF‐α by macrophages.^25^, ^26^

In addition, the phosphorylation of ANXA2 exerts a very important effect on its biological function. The phosphorylation sites of ANXA2 are mainly located at the N‐terminus, and the three most important phosphorylation sites are Ser11,^27^ Ser25^28^ and Tyr23.^29^ The phosphorylation of intracellular ANXA2 is influenced by several factors, and it has been shown that oxidative stress increases intracellular reactive oxygen species (ROS) levels and promotes intracytoplasmic ANXA2 Tyr^23^ phosphorylation.^30^ After Tyr^23^ phosphorylation of intracytoplasmic ANXA2 occurs, it binds to fibrous actin and is subsequently released into the extracellular space.^31^, ^32^ According to these findings, Tyr^23^ phosphorylation and the release of ANXA2 are strongly related, whereas ox‐LDL stimulates macrophages to form foam cells mostly via producing huge quantities of ROS.^33^ In our previous study, we found that PSRC1 could play a role in delaying AS development by inhibiting the inflammatory response of macrophages. In the current research, we found the interaction between PSRC1 and ANXA2 in ox‐LDL‐stimulated macrophages was increased, and a decrease in the ANXA2 content of cell culture supernatants when PSRC1 was overexpressed, suggesting a decrease in the extracellular secretion of ANXA2. We also studied CAD and non‐CAD patients and examined their serum ANXA2 levels; we found that ANXA2 was markedly higher in CAD patients than in non‐CAD patients, presenting that the development of CAD may be closely correlated with ANXA2 levels, and ANXA2 may be a new predictor.

The transcription factor SP1 is a member of the SP/KLF family and is localized in the nucleus. The expression of numerous genes in mammalian cells is regulated by SP1, which is extensively expressed in vivo. Several proteins, including other transcription factors and epigenetic regulators, can interact with SP1. Studies have revealed that SP1 performs many important functions in addition to being a regulator of housekeeping genes.^34^ As a widely available transcription factor, SP1 is crucial for cell development, differentiation, and metabolism.^35^ SP1 mainly binds to the GC box in the promoter region of downstream genes to exert its transcriptional regulatory effects.^36^ In this study, we found that SP1 inhibits PSRC1 expression, and the SP1 inhibitor Mith elevates PSRC1 levels in macrophages; additionally, we found that Mith attenuates the inflammatory response and delays the progression of AS in animal experiments. In the 1950s, Mith was initially classified as an anticancer medication and used to treat testicular cancer and chronic granulomatous leukemia in patients.^37^, ^38^ Numerous studies have confirmed the anti‐tumour effects of Mith on prostate, cervical, lung, oesophageal and breast cancers.^39^, ^40^, ^41^, ^42^ However, due to the high number of adverse effects associated with Mith treatment, novel targeted therapies for tumour treatment have emerged, and Mith has been gradually withdrawn from clinical use in recent years. In animal studies of breast cancer, mice were treated with .3 mg/kg Mith,^42^ and in chemotherapy‐resistant colorectal cancer, 1.5 mg/kg Mith was administered to mice.^43^ In the present study, mice were intraperitoneally treated with .15 mg/kg Mith, which was a lower dose than that used in tumour treatment, and AS was successfully ameliorated. This suggests that treatment of AS with a low dose of Mith may achieve clinical remission with reduced toxic effects.

However, this study still has several limitations. On the one hand, there were patient variations and a small sample size in experiments employing patient monocytes; on the other hand, the flaws in the experimental design and the patient enrollment led to discontinuous and lengthy gaps in the collection of clinical samples, and we will also standardize the collection of clinical samples in the follow‐up study.

In summary, this study reveals the important regulatory role of PSRC1/ANXA2 in AS, and we also found that the SP1 inhibitor Mith mitigates the inflammatory response and delays the progression of AS via PSRC1/ANXA2. These results offer a prospective target and therapeutic agent for the clinical treatment of ASCVD and once again emphasize the important role of the inflammatory response and anti‐inflammatory treatments in the development and treatment of AS.

## CONFLICT OF INTEREST STATEMENT

The authors report no relationships that could be construed as a conflict of interest.

## Supporting information

Supporting Information
