# Supplementary material for: Proline/serine‐rich coiled‐coil protein 1 inhibits macrophage inflammation and delays atherosclerotic progression by binding to Annexin A2
Source: Clin Transl Med. 2023 Mar 17;13(3):e1220. doi: 10.1002/ctm2.1220 (PMC10023832; doi:10.1002/ctm2.1220)
Supplement: Supplementary file 1 — Supporting Information [file CTM2-13-e1220-s001.doc]

**PSRC1 inhibits macrophage inflammation and delays atherosclerosis progression by binding to ANXA2**

Hangyu Pan^1^, Zhongzhou Guo^2^, Ping Lv^3^, Kexin Hu^1^, Tongwei Wu^4^, Yazhi Xue^5^, Yanan Zhang^1#^, Zhigang Guo^6#^

**Affiliations:**

1. Department of Cardiology, State Key Laboratory of Organ Failure Research, Nanfang Hospital, Southern Medical University, Guangzhou 510515, China

2. Department of Pharmacy, Zhujiang Hospital, Southern Medical University, Guangzhou 510280, China

3. Department of Cardiovascular Surgery, Nanfang Hospital, Southern Medical University, Guangzhou 510515, China

4. Department of Medicine Ultrasonics, Nanfang Hospital, Southern Medical University, Guangzhou 510515, China

5. Department of General Practice, Nanfang Hospital, Southern Medical University, Guangzhou 510515, China

6. Department of Cardiology, Huiqiao Medical Center, Nanfang Hospital, Southern Medical University, Guangzhou 510515, China

**Correspondence:**

Zhigang Guo, Ph.D, Department of Cardiology, Huiqiao Medical Center, Nanfang Hospital, Southern Medical University, Guangzhou 510515, China. Tel:(86)020-61642251, Email:guozhigang126@smu.edu.cn

Or

Yanan Zhang, Ph.D, Department of Cardiology, State Key Laboratory of Organ Failure Research, Nanfang Hospital, Southern Medical University, Guangzhou 510515, China. Tel:(86)020-62787094, Email:zyncjdsg@126.com

**Caption for supplementary material**

**Supplementary tables**

Table S1: Logistic regression analysis of the risk factors of CAD

Table S2: The area under the ROC curve of the CAD predicted by indicators

Table S3: All the primers used in the qPCR analysis.

**Supplementary figures**

Fig. S1: Representative peptides of ANXA2 protein.

Fig. S2: ANXA2 affects lipid levels.

Fig. S3: ANXA2 affects the inflammatory response in vivo.

Fig. S4: Generation of PSRC1-/-ApoE-/-mice.

Fig. S5: Validating the efficiency of AAV-shANXA2 injection.

Fig. S6: PSRC1 affects lipid levels in serum via ANXA2.

Fig. S7: PSRC1 affects the inflammatory response in vivo via ANXA2.

Fig. S8: ANXA2 does not affect ERK and P38.

Fig. S9: ANXA2 promotes STAT3 phosphorylation.

Fig. S10: ANXA2 does not affect the expression of lipid-associated proteins.

Fig. S11: The other SPs had no effect on the transcription of PSRC1.

Fig. S12: Mith reduces macrophage inflammatory factor levels.

Fig. S13: Mith does not affect the expression of lipid-associated proteins.

Fig. S14: Mith affects the inflammatory response but not lipid levels in vivo via PSRC1.

**Supplemental Figure 1. Representative peptides of ANXA2 protein.** (A) ANXA2 protein identification mass spectrometry peak map. (B) The full-length sequence of ANXA2 protein. Red: peptide shown in Figure S1A. Green: other peptides identified by mass spectrometry. Black: unidentified sequences.

**Supplemental Figure 2. ANXA2 affects lipid levels.** (A-B) Representative images and statistical graph of aortic bulk plaques in the Ad-GFP and Ad-shANXA2 groups after 8 weeks of chow diet feeding. (C) Body weight of 2 groups of mice. TG (D), CHOL (E), LDL (F), and HDL (G) levels in the serum of the Ad-GFP and Ad-shANXA2 groups. n=6 per group, Student's t test, ***P<0.001,****P<0.0001, ns P>0.05.

**Supplemental Figure 3. ANXA2 affects the inflammatory response in vivo.** ELISA for ANXA2 (A), IL-1β (B) and IL-6 (C) levels in serum. n=6 per group, Student's t test, ***P<0.001,****P<0.0001.

**Supplemental Figure 4. Generation of PSRC1^-/-^ApoE^-/-^mice.** (A) Knockout of PSRC1 exon 4 using CRISPR‒Cas9. (B) PSRC1 protein sequence. Red: amino acid sequence of the deleted exon. Agarose gel electrophoresis was used to examine the effect of PSRC1 (C) and ApoE (D) knockout.

**Supplemental Figure 5. Validating the efficiency of AAV-shANXA2 injection.** ANXA2 mRNA levels in peritoneal macrophages (A) and livers (B) of the AAV-GFP and AAV-shANXA2 groups. n=6 per group, Student's t test, ***P<0.001, ns P>0.05. Cross-sections of the mouse atherosclerotic aortic roots were stained with immunofluorescence. (C) GFP in the F4/80+ area of atherosclerotic aortic root sections. (D) Representative immunofluorescence images demonstrating the distribution of PSRC1 and ANXA2 in atherosclerotic aortic root sections.

**Supplemental Figure 6. PSRC1 affects lipid levels in serum via ANXA2.** (A) Body weight of 3 groups of mice. TG (B), CHOL (C), LDL (D), and HDL (E) levels in the serum of the 3 groups. n=6 per group, one-way ANOVA, *P<0.05,**P<0.01, ***P<0.001, ****P<0.0001, ns P>0.05.

**Supplemental Figure 7. PSRC1 affects the inflammatory response in vivo via ANXA2.** ELISA for ANXA2 (A), IL-1β (B) and IL-6 (C) levels in the serum of 3 groups. n=6 per group, one-way ANOVA, *P<0.05,**P<0.01, ***P<0.001, ****P<0.0001.

**Supplemental Figure 8. ANXA2 does not affect ERK and P38.** Raw264.7 cells transfected with si-NC and si-ANXA2 were treated with ox-LDL for 24 h. Western blotting was performed to measure p-ERK1/2, ERK1/2, p-P38 and P38 expression levels (A) and p-ERK1/2/ERK1/2 and p-P38/P38 were quantified (B-C), n=3 per group, Student's t test, nsP>0.05.

**Supplemental Figure 9. ANXA2 promotes STAT3 phosphorylation.** Cross-sections of AAV-GFP and AAV-shANXA2 mouse atherosclerotic aortic roots were stained with immunofluorescence for ANXA2 and p-STAT3.

**Supplemental Figure 10. ANXA2 does not affect the expression of lipid-associated proteins.** Raw264.7 cells and AML12 cells were transfected with si-NC or si-ANXA2 for 30 h. The mRNA levels of ABCA1 (A), ABCG1 (B), LXR (C), SRB1 (D), SREBP2 (E) in Raw264.7 and PCSK9 (F) in AML12 were measured by RT‒qPCR. n=3 per group, Student’s t test, nsP>0.05.

**Supplemental Figure 11. The other SPs had no effect on the transcription of PSRC1.** Raw264.7 cells were transfected with si-NC and si-SP2(A), si-SP3(B), si-SP4(C) and si-SP8(D) for 30 h, and PSRC1 mRNA was measured by RT-qPCR, n=3 per group, Student’s test, ns P>0.05.

**Supplemental Figure 12. Mith reduces macrophage inflammatory factor levels.** Raw264.7 cells were treated with PBS or Mith for 24 h and then stimulated with ox-LDL for 24 h. The mRNA levels of IL-1β (A), IL-6 (B),TNF-α (C), IL-4 (D), TGF-β (E) and IL-10 (F) were measured by RT‒qPCR. IL-1β (G), IL-6 (H) and ANXA2 (I) in cell culture supernatants were measured by ELISA. n=3 per group, Student’s t test, *P<0.05, **P<0.01, ns P>0.05.

**Supplemental Figure 13. Mith does not affect the expression of lipid-associated proteins.** Raw264.7 cells and AML12 cells were treated with PBS or Mith for 24 h and then stimulated with ox-LDL for 24 h. The mRNA levels of ABCA1 (A), ABCG1 (B), LXR (C), SRB1 (D) and SREBP2 (E) in Raw264.7 cells and PCSK9 (F) in AML12 cells were measured by RT‒qPCR. n=3 per group, Student’s t test, **P<0.01, nsP>0.05.

**Supplemental Figure 14. Mith affects the inflammatory response but not lipid levels *in vivo* via PSRC1.** (A) Body weight of 4 groups of mice. TG(B), CHOL(C), LDL(D) and HDL(E) levels in the serum of the 4 groups. ANXA2 (F), IL-1β (G) and IL-6 (H) levels in the serum of the 4 groups measured by ELISA. n=6 per group, one-way ANOVA, *P<0.05,**P<0.01, ***P<0.001, ****P<0.0001, ns P>0.05.

**Supplemental Figure 15. Mith reduces the release of ANXA2 in THP-1 cell line.** THP -1 cells were divided into two groups and stimulated with PBS or 200 nM Mith for 24h followed by 100ug/ml ox-LDL for 24h. (A) ANXA2 level in cell cultured supernatants were detected by ELISA (n=4). (B) ANXA2 mRNA was detected by RT-qPCR (n=6). Student’s t test, ***P<0.001, nsP>0.05.

**Supplemental Table 1. Logistic regression analysis of the risk factors of CAD**

| **Factor** | **Single factor logistic regression analysis** | | **Multivariable logistic regression analysis** | |
| --- | --- | --- | --- | --- |
|  | **OR(95%CI)** | **P-value** | **OR(95%CI)** | **P-value** |
| **ANXA2** | 1.064(1.033-1.095) | 0.00003* | 1.059(1.027-1.092) | 0.002* |
| **Sex** | 3.051(1.756-5.300) | 0.000076* | 2.040(1.057-3.936) | 0.033* |
| **Diabetes** | 2.429(1.271-4.641) | 0.007* | 2.072(1.007-4.261) | 0.048* |
| **Leukocyte** | 1.244(1.096-1.412) | 0.001* | 1.205(1.047-1.385) | 0.009* |
| **Creatinine** | 1.018(1.006-1.029) | 0.003* | 1.014(1.001-1.026) | 0.028* |
| **Age** | 1.001(0.972-1.031) | 0.937 |  |  |
| **BMI** | 1.030(0.944-1.125) | 0.505 |  |  |
| **Smoke** | 2.249(1.298-3.898) | 0.004* |  |  |
| **Hypertension** | 1.405(0.833-2.371) | 0.203 |  |  |
| **Cholesterol** | 1.210(0.975-1.503) | 0.084 |  |  |
| **Triglycerides** | 1.119(0.900-1.391) | 0.311 |  |  |
| **HDL-C** | 0.801(0.515-1.247) | 0.326 |  |  |
| **LDL-C** | 1.234(0.968-1.573) | 0.089 |  |  |
| **CRP** | 1.024(0.993-1.056) | 0.127 |  |  |

CRP: C-reative protein. HDL-C: high density lipoprotein-cholesterol. LDL-C: low density lipoprotein-cholesterol. *P<0.05

**Supplemental Table 2. The area under the ROC curve of the CAD predicted by indicators**

| **Variable** | **Area** | **Std. Error** | **Sig.** | **95%CI** |
| --- | --- | --- | --- | --- |
| **CRP** | 0.610 | 0.036 | 0.004 | 0.540-0.680 |
| **ANXA2(ng/ml)** | 0.671 | 0.033 | 0.000008 | 0.606-0.736 |
| **ANXA2*CRP** | 0.668 | 0.034 | 0.000011 | 0.602-0.734 |

CRP: C-reative protein. ANXA2*CRP:ANXA2 combined with CRP

**Supplemental Table 3. Primers**

| Primers name | | Sequence (5'~ 3') |
| --- | --- | --- |
| Mouse IL-1β | Forward Primer | CACTACAGGCTCCGAGATGAACAAC |
|  | Reverse Primer | TGTCGTTGCTTGGTTCTCCTTGTAC |
| Mouse IL-6 | Forward Primer | CTTCTTGGGACTGATGCTGGTGAC |
|  | Reverse Primer | TCTGTTGGGAGTGGTATCCTCTGTG |
| Mouse TNF-α | Forward Primer | ATGTCTCAGCCTCTTCTCATTC |
|  | Reverse Primer | GCTTGTCACTCGAATTTTGAGA |
| Mouse ANXA2 | Forward Primer | TGCCTACGGGTCAGTCAAAC |
|  | Reverse Primer | GCCCAAAATCACCGTCTCC |
| Mouse β-actin | Forward Primer | GTGCTATGTTGCTCTAGACTTCG |
|  | Reverse Primer | ATGCCACAGGATTCCATACC |
| Human ANXA2 | Forward Primer | GGATATTGCCTTCGCCTACCAGAG |
|  | Reverse Primer | GCGTCATACTGAGCAGGTGTCTTC |
| Human β-actin | Forward Primer | CCTTCCTGGGCATGGAGTC |
|  | Reverse Primer | TGATCTTCATTGTGCTGGGTG |
| Mouse IL-4 | Forward Primer | TACCAGGAGCCATATCCACGGATG |
|  | Reverse Primer | TGTGGTGTTCTTCGTTGCTGTGAG |
| Mouse IL-10 | Forward Primer | TTCTTTCAAACAAAGGACCAGC |
|  | Reverse Primer | GCAACCCAAGTAACCCTTAAAG |
| Mouse TGF-β | Forward Primer | CCAGATCCTGTCCAAACTAAGG |
|  | Reverse Primer | CTCTTTAGCATAGTAGTCCGCT |
| Mouse ABCA1 | Forward Primer | AGAAGGAGGCTCGGCTGAAGG |
|  | Reverse Primer | GAGGGATGAGGCTGCTAACAAACC |
| Mouse ABCG1 | Forward Primer | CATGCTGCTGCCTCACCTCAC |
|  | Reverse Primer | TCTCGTCTGCCTTCATCCTTCTCC |
| Mouse LXR | Forward Primer | AACTGAAGCGGCAAGAAGAGGAAC |
|  | Reverse Primer | TGGCAGGACTTGAGGAGGTGAG |
| Mouse SRB1 | Forward Primer | GCAAATTTGGCCTGTTTGTT |
|  | Reverse Primer | GATCTTGCTGAGTCCGTTCC |
| Mouse SREBP2 | Forward Primer | CCGCCTCCTTCTCCTTCTCCTC |
|  | Reverse Primer | CCGCAGTTTCACCATAGCCAGAG |
| Mouse PCSK9 | Forward Primer | AGCAGCCAGGTGGAGGTGTATC |
|  | Reverse Primer | GCCTGTCTGTGGAAGCGTGTC |
